# Photothermal Circular Dichroism Measurements of Single Chiral Gold Nanoparticles Correlated with Electron Tomography

**DOI:** 10.1021/acsphotonics.2c01457

**Published:** 2022-11-08

**Authors:** Patrick Spaeth, Subhasis Adhikari, Wouter Heyvaert, Xiaolu Zhuo, Isabel García, Luis M. Liz-Marzán, Sara Bals, Michel Orrit, Wiebke Albrecht

**Affiliations:** † Huygens-Kamerlingh Onnes Laboratory, Leiden University, 2300Leiden, RA, The Netherlands; ‡ Department of Sustainable Energy Materials, AMOLF, Science Park 104, 1098Amsterdam, XG, The Netherlands; § EMAT and NANOlab Center of Excellence, University of Antwerp, Groenenborgerlaan 171, B-2020Antwerp, Belgium; ∥ Basque Research and Technology Alliance (BRTA), CIC biomaGUNE, Paseo de Miramón 182, 20014Donostia-San Sebastián, Spain; ⊥ CIBER de Bioingeniería, Biomateriales y Nanomedicina (CIBER-BBN), Paseo de Miramón 182, 20014Donostia-San Sebastián, Spain; ○ School of Science Engineering, The Chinese University of Hong Kong (Shenzhen), Shenzhen518172, China; △ Ikerbasque (Basque Foundation for Science), 48009Bilbao, Spain

**Keywords:** photothermal microscopy, chirality, plasmonic nanoparticles, 3D characterization, helical morphology

## Abstract

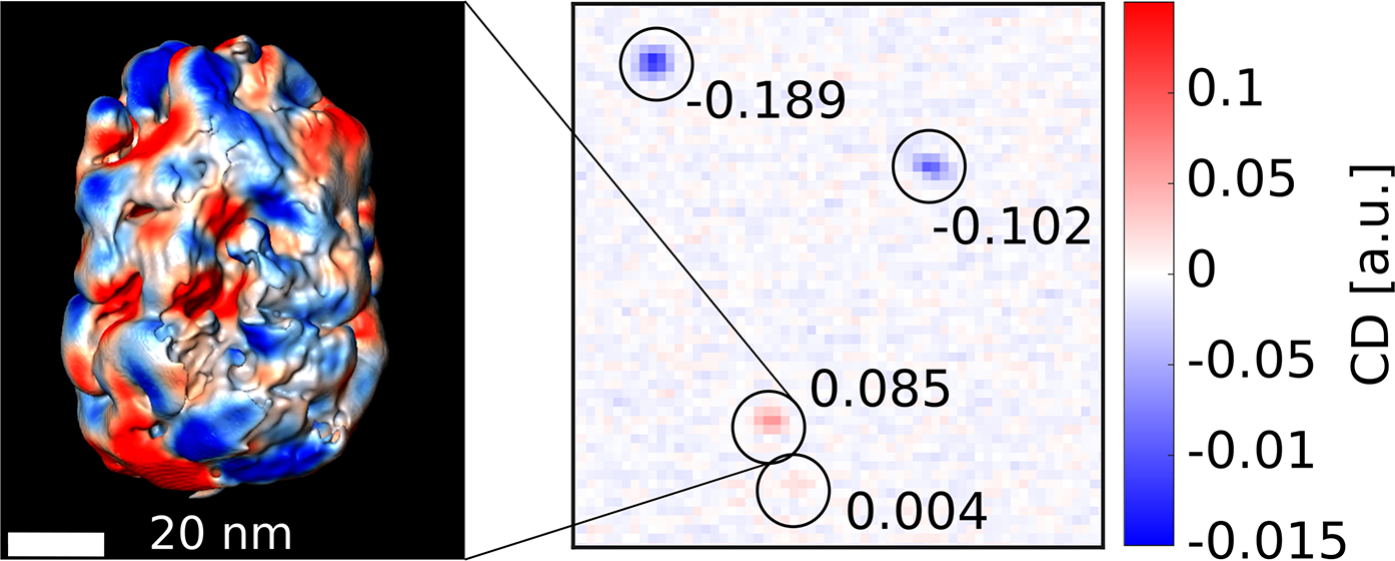

Chemically synthesized metal nanoparticles with morphological chiral features are known to exhibit strong circular dichroism. However, we still lack understanding of the correlation between morphological and chiroptical features of plasmonic nanoparticles. To shed light on that question, single nanoparticle experiments are required. We performed photothermal circular dichroism measurements of single chiral and achiral gold nanoparticles and correlated the chiroptical response to the 3D morphology of the same nanoparticles retrieved by electron tomography. In contrast to an ensemble measurement, we show that individual particles within the ensemble display a broad distribution of strength and handedness of circular dichroism signals. Whereas obvious structural chiral features, such as helical wrinkles, translate into chiroptical ones, nanoparticles with less obvious chiral morphological features can also display strong circular dichroism signals. Interestingly, we find that even seemingly achiral nanoparticles can display large *g*-factors. The origin of this circular dichroism signal is discussed in terms of plasmonics and other potentially relevant factors.

## Introduction

Chiral plasmonic nanoparticles (NPs) have recently received much attention owing to their potential in light manipulation as, for example, in circular polarizers,^1^ enantiomeric sensing of biomolecules,^2^ chiral waveguides,^3^ or negative refractive index materials.^4^ Although top-down fabricated plasmonic NPs can be tailor-made with specific optical properties,^5^ their fabrication process is difficult for up-scaling. Wet-chemical synthetic processes can be up-scaled more easily and significant progress has been recently made in the solution-based synthesis of chiral plasmonic NPs,^6−9^ but precisely controlling the NP shapes and hence the chiral features remains a difficult task. This is not only limited by complicated synthesis procedures but also by our limited understanding of the precise relation of structural and morphological features that effectively translate into a desired chiroptical response.

The standard way of determining the chiroptical response is by measuring a circular dichroism (CD) spectrum using a commercial CD spectrometer. CD is the differential absorption of left- and right-circularly polarized light and a typical CD spectrometer measures the CD spectrum, via extinction, of an ensemble of molecules or particles, averaging out heterogeneous single-molecule/-particle information. To overcome this limitation, several research groups have developed single-particle circular dichroism techniques.^10−14^ We have recently developed photothermal circular dichroism microscopy (PT CD),^15^ which is capable of measuring the direct differential absorption, free from scattering contributions, and have demonstrated unprecedented sensitivity.^16^ Specifically, by employing a dual modulation of the polarization, we successfully reject leakage of linear dichroism into the CD signal, which enables us to measure anisotropy factors, so-called *g*-factors, as low as 10^–3^ and even smaller.

Such single-particle CD techniques are imperative for achieving a better understanding of the structure-chiroptics relation of chiral plasmonic NPs, specifically if they can be correlated to actual structural information on a single-NP level. Recently, González-Rubio et al. strove to gain a deeper understanding of this correlation by advanced electron tomography (ET) analysis of quasi-helical gold (Au) nanorods (NRs).^17^ By applying fast Fourier transformations on the 3D reconstructions of the NPs, they concluded that repetitive wrinkles, that is, helical features, caused strong chirality in these systems. However, this analysis was not coupled to single-particle CD measurements. In fact, recent single-particle scattering-based CD measurements performed by Karst and co-workers questioned whether geometrical chiral features really cause the expected chiroptical response.^7^ The authors found that apparently similar helicoid NPs exhibited different chiroptical responses in strength and spectral dependence. They also attempted to correlate the optical response to the NPs’ morphologies by scanning electron microscopy (SEM), but they could not identify any obvious structural differences that would lead to the observed spread in chiroptical response. However, these authors were limited by the low spatial resolution of their SEM measurements and the inaccessibility of the complete 3D morphological information. To obtain a deeper understanding of complex chiral NPs, 3D morphological analysis at the single particle level is necessary. The latter can be retrieved by electron tomography and we have shown that morphological helical features can be identified with advanced analysis methods from ET reconstructions.^18^

Here, we combine our sensitive optical PT CD measurements with electron tomography performed by high-angle annular dark-field scanning transmission electron microscopy (HAADF-STEM) on single chiral NRs synthesized according to González-Rubio et al.^17^ We observe strong particle-to-particle differences in the strength of the chiroptical signal, which can only partially be explained by morphological differences. We therefore extend our correlative measurements to seemingly achiral gold NPs and perform boundary element method (BEM) simulations to decouple plasmonic from other effects.

## Results and Discussion

We started by synthesizing a batch of chiral gold nanorods, hereafter referred to as batch 1, consisting of two samples with opposite handedness to each other, through the addition of either *R*- or *S*-BINAMINE. Their detailed synthesis parameters can be found in the Experimental Section. Their ensemble CD measurements are presented in Figure S1a and HAADF-STEM images of batch 1 are shown in Figure S2. Orthoslices, which are slices through 3D reconstructions obtained from ET of two representative particles are shown in Figure S3. From Figures S2 and S3 it can be seen that batch 1 has sharp helical wrinkles, which were earlier identified as the origin for the strong chiroptical response of these nanorods.^17^

For the optical measurements of particles from batch 1, we prepared two samples, one for each handedness of the chirality encoder molecule (*R* and *S* enantiomers of BINAMINE), by spin-coating the respective dispersion (*R* or *S*) on a cleaned glass cover slide that was previously rendered hydrophilic by ozone cleaning, and performed single-particle PT and PT CD measurements of >100 particles for each sample. Typical PT CD images, from which we determined the chiroptical handedness and anisotropy factors *g* are shown in Figure S4. All experimental details are described in the Experimental Section and in our previous publications.^15,16^ In short, PT imaging is purely sensitive to the absorption of an object and therefore the PT images show where absorbing particles are located. The strength of the PT signal scales linearly (to first approximation) with the strength of the absorption, i.e. the absorption cross section for metal NPs. PT CD is a recent modification to the normal PT technique, which instead of absolute absorption measures the difference of absorption between left and right circularly polarized light (LCP/RCP) through modulation of the incoming polarization. Red and blue color in the PT CD images refer to more absorption of LCP and RCP of an object, respectively. The corresponding *g*-factors are then calculated by dividing the PT CD by the pure PT signal (more details are in the Experimental Section).

**Figure 1:**
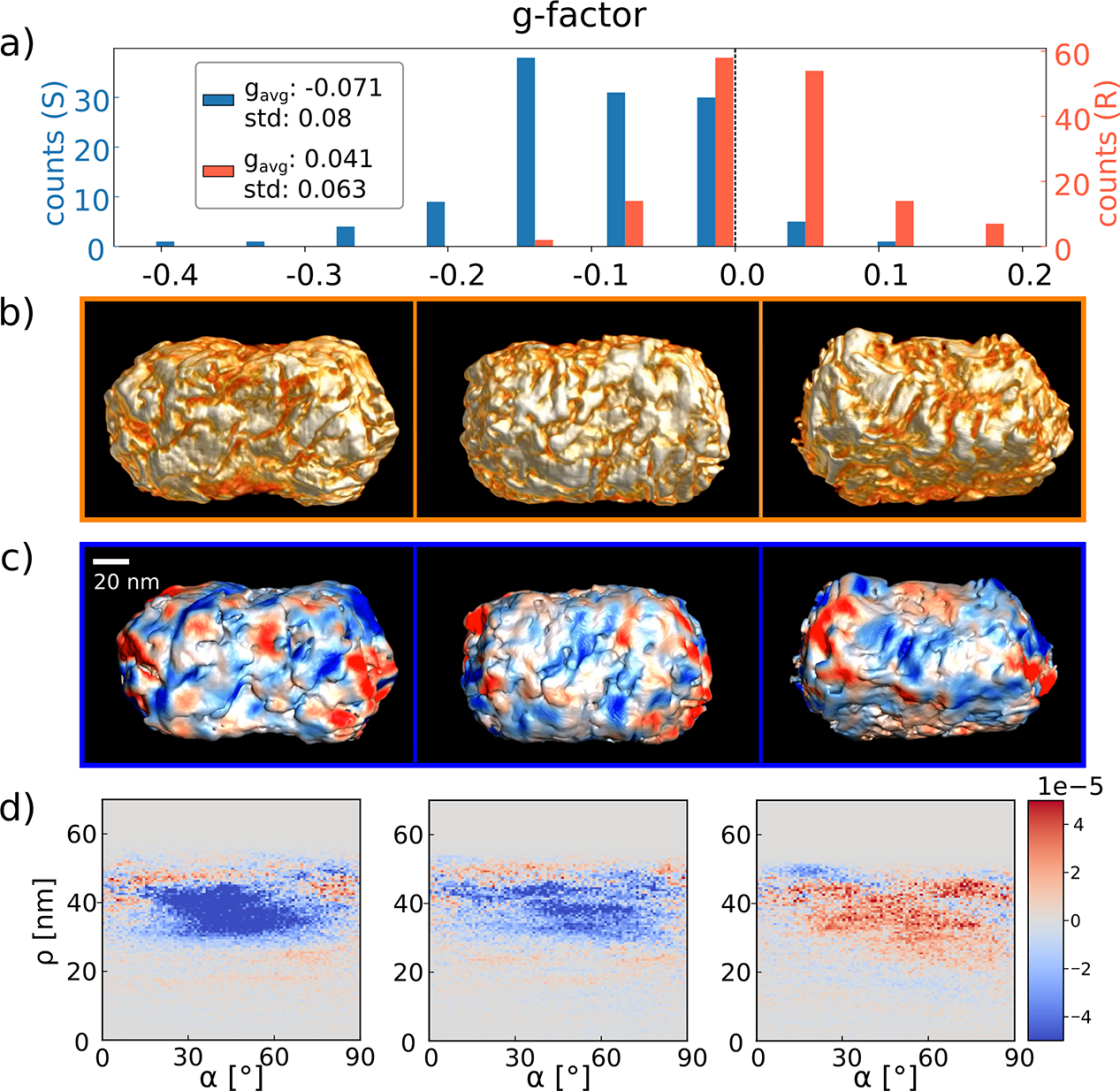
PT CD and ET measurements of the NRs from batch 1. (a) Histogram of *g*-factors of the *R* (in red) and *S* version (in blue) of batch 1. (b) 3D visualizations of ET reconstructions of three NRs and (c) the corresponding 3D helicity maps. The left two NRs are from the *S* sample and the right NR is from the *R* sample. The scale bar is 20 nm. (d) Helicity functions of the three NRs, which display helical features averaged over a cylindrical slice for a given radius ρ and feature inclination angle α. (c, d) Red (positive helicity) and blue (negative helicity) colors correspond to right- and left-handed helical features, respectively.

Figure 1a shows the histograms of *g*-factors for the *R* and *S* enantiomers of the chiral nanoparticles in red and blue, respectively. Although both samples display a broad distribution of *g*-factors, exhibiting positive and negative *g*-factors for both the *R* and the *S* versions, both histograms can be clearly distinguished with an obvious bias toward one *g*-factor sign or the other. In addition, on a single nanoparticle level the *g*-factors can be very high, with values up to −0.4 and 0.2 for the *S* and *R* enantiomers, respectively. It is also worth noting that the sign of the average *g*-factors, determined from the histograms of our single-particle measurements, agree well with the ensemble solution-based measurements shown in Figure S1, but the average values, −0.071 and 0.041, for the *S* and *R* versions, respectively, are significantly higher than their ensemble counterparts (±0.015). We speculate that three possible reasons might be responsible for this difference. An obvious difference between the ensemble and single-particle measurements is the surrounding dielectric environment. Whereas the ensemble measurements are performed in aqueous solution, isolated nanoparticles are lying on a solid support surrounded by immersion oil. Our immersion oil has a similar refractive index (1.34) to that of water (1.33), but the glass substrate (1.51) raises the effective surrounding dielectric constant. In addition, we measure pure absorption contributions, free of scattering, whereas a standard CD spectrometer measures extinction (including both absorption and scattering). Another difference is the orientation of the nanorods. In solution, all possible particle orientations are averaged, whereas the nanorods are lying flat on the support in our single-particle measurements. This bias in orientation could be one reason why we see an increase in *g*-factor values at our measurement wavelength of 660 nm. The clear distinction in *g*-factor histograms for the different enantiomers in batch 1 and the observed structural chiral features, that is, wrinkles (Figures S2 and S3) are in good agreement with the earlier observations by González-Rubio et al.^17^ that the helical morphological features cause the chiroptical signal. To further quantify this correlation, we performed electron tomography on the *R* and *S* enantiomers of batch 1. 3D visualizations of reconstructions of three NRs are shown in Figure 1b. Next, we applied the method recently reported by Heyvaert et al.^18^ to analyze the helical features from these reconstructions. Figure 1c shows the 3D helicity maps, which are created by calculating a helicity measure for a small window around each voxel in the particle (details in Experimental Section). As also observed in our earlier work,^18^ every particle displayed both, left- and right-handed local chiral features. However, one handedness clearly dominates in each particle, as seen from the helicity function plots in Figure 1d. Here, the helicity function, our measure of the strength and handedness of helical features, is plotted as a function of radius ρ and inclination angle α of the helical features. The plots tell us that the helical features were located in the shell of the nanorods (30 nm < ρ < 50 nm), which is expected because the core is achiral. In addition, it can be seen that the helical wrinkles are spread over a wide range of inclination angles up to 90°. For the particles analyzed in ref (18), the inclination angle was limited to a smaller range (mostly below 45°), indicating more order in the helical wrinkles. Since the average *g*-factor of nanorods reported in refs (17) and (18) was higher compared to the particles studied in this work, we believe that the spread of helical wrinkles over larger inclination angles led to a decrease in the chiroptical response. Nonetheless, from Figure 1 and the cited earlier work it is clear that a dominant bias in structural helical features leads to chiroptical signals with the expected handedness, that is, left- and right-handed helical features result in negative and positive *g*-factors, respectively.

**Figure 2:**
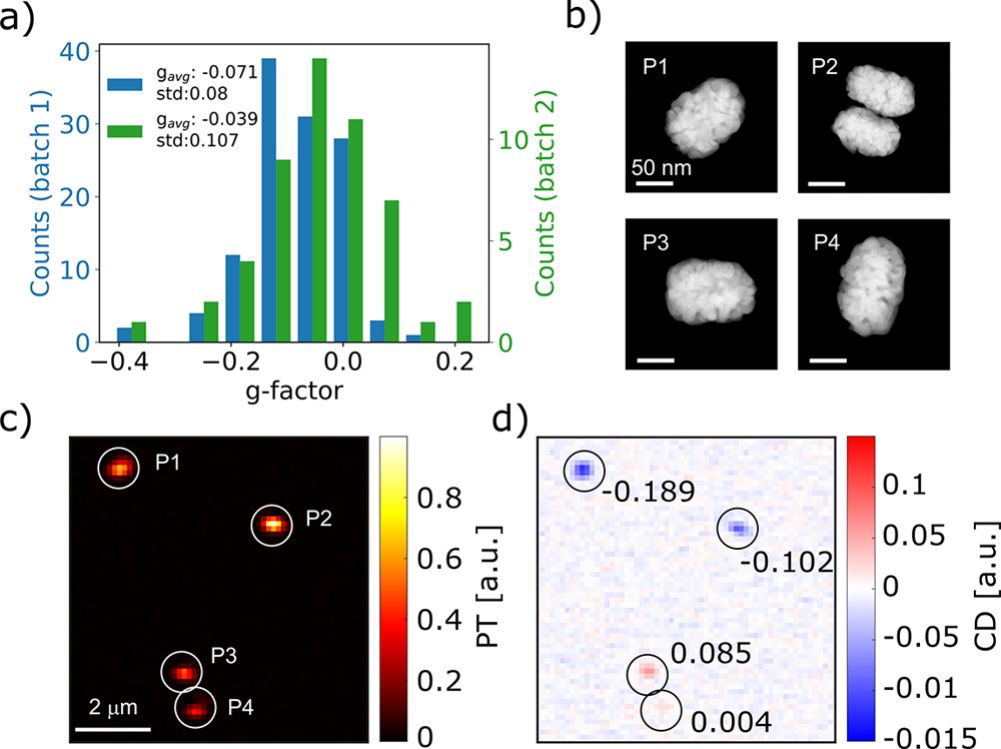
(a) Comparison of *g*-factor histograms for the two different NR batches, in blue batch 1 and green batch 2. Both histograms correspond to the *S* enantiomer of each batch. The average *g*-factor and the width of the distributions, that is, their standard deviations, are indicated. (b–d) Correlated HAADF-STEM and PT CD measurements. (b) HAADF-STEM images of three single NRs and one dimer from batch 2 as labeled in (c), which shows the corresponding photothermal measurement. (d) PT CD measurement corresponding to the same region, with the corresponding *g*-factors.

Sample degradation can lead to a loss of such clear helical wrinkles.^18^ The loss of specific structural features provides us with an opportunity to investigate the importance of such features with respect to the optical properties. We observed such a loss of the helical wrinkles for a second sample batch, referred to as batch 2, which was synthesized under slightly different conditions (see Experimental Section for details). HAADF-STEM images and orthoslices through ET reconstructions of batch 2 are shown in Figures S3 and S5. The corresponding ensemble CD spectra of the *R* and *S* versions of batch 2 are shown in Figure S1b. Compared to batch 1, helical wrinkles appear to be less present for batch 2. We believe that this morphological difference between the two batches can be attributed to changes in storage conditions (surfactant concentration,^17^ exposure to air during the preparation of TEM grids) and heating (especially under the photothermal measurements), which is part of an ongoing study. However, the difference in structural features is not reflected in the g-factor strength of the ensemble CD spectra (Figure S1). In fact, at our measurement wavelength of 660 nm for the PT CD measurements both samples exhibit similar anisotropy factors around ±0.015. Nonetheless, a clear difference in single-particle *g*-factors can be seen from the PT CD histograms. Figure 2 compares the histograms of the *S* version of batch 1 (blue) and batch 2 (green). The measured *g*-factors of batch 2 display a broader distribution including high values of opposite handedness (see discussion on the sign of the CD signal for batch 2 in the Supporting Information), resulting in a lower average *g*-factor compared to batch 1 and a larger standard deviation.

Interestingly, however, the particles of batch 2 contained a significantly less clear helical motif (Figures S3 and S5) and yet a non-negligible amount of single particles exhibited strong *g*-factors with values above ±0.2. To shine more light on the connection between structure and chirality, we therefore performed correlated HAADF-STEM and PT CD measurements of these particles. For these correlative measurements, we dropcasted the solution on a Ted Pella TEM grid with 18 nm thick electron-transparent SiO_2_ windows, which was rendered hydrophilic previously by an ozone cleaning step. HAADF-STEM images of four particles, including one dimer, are exemplarily shown in Figure 2 alongside their PT and PT CD images. For all particles, different handedness and strengths of *g*-factors can be observed. A total of 25 additional measured particles, their *g*-factors, and HAADF-STEM images are shown in Figure S5.

**Figure 3:**
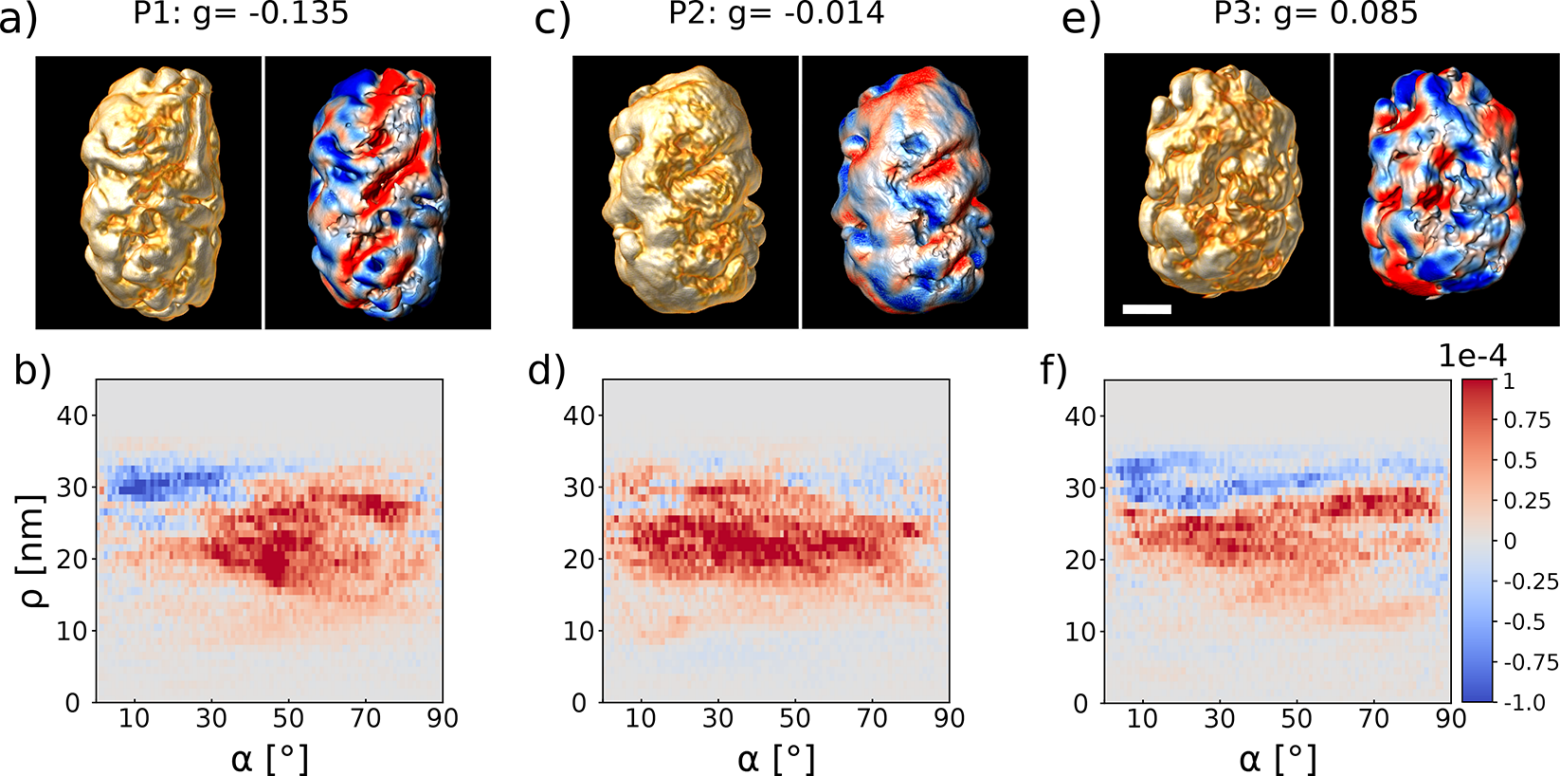
Correlation of measured *g*-factors and ET on single chiral NRs from the *S* enantiomer of batch 2. The left panels in (a), (c), and (e) show 3D visualizations of the ET reconstructions of three nanorods with different *g*-factors, which were measured by single-particle PT CD. The scale bar is 20 nm. The right images in panels (a), (c), and (e) show the corresponding 3D helicity maps of the same nanorods. Red and blue colors correspond to positive (right-handed) and negative (left-handed) helicities. (b), (d), and (f) display the corresponding helicity as a function of radius ρ and inclination angle α.

For seven of the particles shown in Figures 2 and S5, we also performed an electron tomography analysis similar to that presented in Figure 1. The left panels in Figure 3a, c, and e show the 3D visualizations of the ET reconstructions for three of those nanorods with significantly different *g*-factors, from a highly chiral NR with a negative *g*-factor (a) to a NR with a one-order-of-magnitude lower *g*-factor (c) and a NR with a strong positive *g*-factor (e). The right panels show the corresponding 3D helicity maps and their helicity functions are plotted in Figures 3b, d and f. The helicity functions for all 7 nanorods and their corresponding *g*-factors are shown in Figure S6. It should be noted that the normalization process used in the analysis prohibits a direct comparison with the values of the structural helicity measured for batch 1. There seems to be no obvious connection between the structural helicity and the measured *g*-factors for the particles in batch 2. In fact, the helicity functions (Figures 3 and S6) show for most particles left and right-handed features, for some NRs (Figure S6) even of similar strength. Nonetheless, many nanorods display strong *g*-factors. For example, particle 1 in Figure 3 has a *g*-factor of −0.135, which is unexpected on the basis of its structural helicity. The helicity function is dominated by right-handed features (red) and only a small area contains left-handed features at low inclination angles (Figure 3b). Similar observations can be made for the other particles. It should be noted, however, that our structural analysis only identifies helicity. Morphological chirality might, of course, also stem from nonhelical features, but the corresponding quantification is not straightforward and novel methods are needed to do so for such complex morphologies. In addition, we measure the optical *g*-factors for one single wavelength (660 nm). Consequently, spectral shifts might hinder a direct comparison. Based on the analysis of the PT and PT CD signals, we believe that spectral shifts seem to be small in our case (see discussion around Figure S7), but in future work we aim to extend our method to a broad wavelength range.

In any case, these measurements raise questions about our understanding of chiroptical activity in complex plasmonic systems and where the spread of single-particle *g*-factors is coming from. Can optical *g*-factors be dominated by the plasmonic enhancement of local structural features such as the left-handed feature at low inclination angles evident from the helicity function of particle 1 (Figure 3)? For nanoparticles with clear structural helicity, as in the case of batch 1, this might not be easily noticeable, as local features are more inclined to have the same handedness. For NPs with less homogeneous structural features and handedness, however, it could significantly influence the chiroptical response. To find possible answers, we decided to remove the helicity parameter and to measure seemingly achiral and uniform quasi-spherical AuNPs, which have displayed surprisingly high CD signals in earlier work.^15^

### Nominally Achiral Nanoparticles

**Figure 4:**
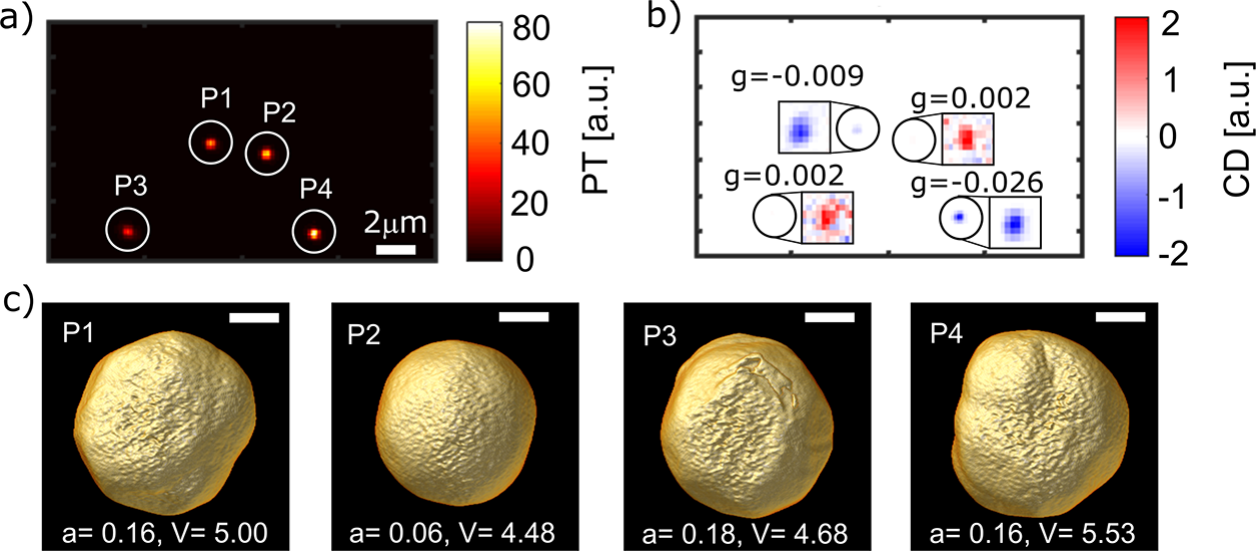
Correlative measurements of seemingly achiral faceted gold NPs. (a) PT and (b) PT CD images of four single gold NPs. Solid circles are drawn to show correlation between the two images. To show the contrast of the weak signals in CD, each particle’s image in (b) is shown with an adjusted contrast as marked with square boxes. The numbers in (b) correspond to the particle’s *g*-factor. (c) 3D visualizations of the ET reconstructions of the four single particles as labeled in (a). The scale bar is 30 nm, *a* is the unitless asphericity, and *V* is the volume of the particle in units of 10^5^ nm^3^.

We compared two samples of nominally achiral NPs (Figure S8): (1) highly isotropic spherical gold NPs with a diameter of 60 nm and (2) faceted single-crystalline NPs of 100 nm size. Both samples were uniform in size and shape (standard deviations of <5% size variation for samples) and served as test samples for analyzing the influence of shape anisotropy. Figure S9 compares the measured *g*-factor histograms of both samples. It should be noted that we used 532 nm excitation instead of 660 nm to match the plasmon resonance of the spherical-like NPs and also used immersion oil of a refractive index of 1.51 as a photothermal medium to match the refractive index of the underlying glass. The first observation is that the averaged measured *g*-factors were 1–2 orders of magnitude lower than those for the NRs and were close to zero (−0.003 ± 0.007 for the faceted NPs and 0.0001 ± 0.002 for the spherical NPs). The latter was particularly true for the spherical NPs, for which the average *g*-factor was one order of magnitude lower, compared to the faceted NPs. To explore why seemingly achiral faceted NPs exhibited higher *g*-factors compared to the nonfaceted spherical ones, we performed correlative PT CD and ET experiments. Figure 4 shows representative measurements of four single gold NPs. Figure 4a,b displays PT and PT CD images and Figure 4c shows the corresponding 3D tomography visualizations of the same four particles in their respective orientations on the substrate. 3D tomography visualizations of all four particles in three orthogonal planes, that is, *XY*, *XZ*, and *YZ* are shown in the Supporting Information (Figure S10). A first observation is that, despite the rather uniform volume of the NPs, claimed by the manufacturer and verified by ET, as shown in Figure S11, the PT signal, which scales linearly with absorption, showed variations in intensity. We believe that this is a consequence of the curved topography of the TEM windows, causing particles to be either in- or slightly out-of-focus within one scan area. We verified that this has no influence on the determination of the *g*-factor as defocusing affects both signals, PT and CD, in the same way (Figure S12).

**Figure 5:**
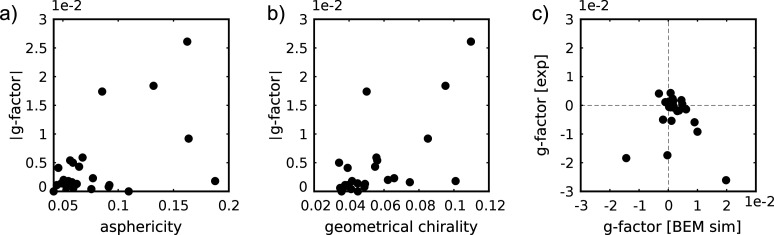
Correlation plots of measured *g*-factors of 25 faceted NPs as a function of (a) NPs’ asphericities, (b) geometrical chiralities, and (c) simulated *g*-factors using the ET reconstructions as input. The *g*-factors were simulated using the boundary element method and extracted at 532 nm.

Although most NPs were rather uniform sphere-like objects with some facets, the 3D reconstructions in Figure 4c demonstrate that more irregular shapes could also be observed. It should be noted that the region shown in Figure 4 was chosen to display differences in NP shapes that could occur. Most of the measured NPs were closer to particle P2 in shape. To understand how much such anisotropies affected the PT CD strength, we determined the asphericities of each NP from the tomography reconstructions and compared it to the measured *g*-factors (details in Experimental Section). The corresponding values for the four NPs in Figure 4 are shown alongside the reconstructions in Figure 4c. Here, an asphericity value of 0 represents perfectly spherical NPs and values >0 indicate nonspherical NPs. For the four particles presented in Figure 4 it can be seen that a low asphericity seems to be related to a low PT CD signal. On the other hand, particles with a larger asphericity value exhibited a larger dispersion of PT CD signals. Both observations are confirmed by Figure 5a, where we summarized the correlative measurements of 25 NPs. Indeed, our findings strongly suggest that asphericity is required to create CD but is not the only prerequisite for a strong CD signal.

One striking observation of Figure 5a is that some of those seemingly achiral NPs exhibited *g*-factors on the order of 10^–2^, which is the same average order of magnitude of the chirality strength of the chiral NRs in batches 1 and 2. To understand whether the relatively high *g*-factors resulted from any structural chirality, we determined the latter based on the tomography reconstructions. Since these particles lack any obvious helical wrinkles or other systematic chiral features it is difficult to categorize them in a meaningful way into left or right handed.^19,20^ Instead, we calculated the geometrical chirality by mirroring the 3D reconstruction and maximizing the overlap between the two mirrored objects by rotation and translation. The mean-square deviation between these maximally overlapped reconstructions can then be used as a measure of the geometrical chirality, where a difference of 0 indicates a fully achiral NP. Figure 5b shows the correlation between this chirality measure and the optical PT CD measurements. Although our statistics were limited due to the complexity of the correlative measurements, NPs with a low geometrical chirality seem to display lower *g*-factors. Interestingly, the observed geometrical chirality is somewhat accidental as the NPs are supposed to be achiral. One origin might be an asymmetric distribution of surface facets. In any case, it shows that structural chiral features can occur unexpectedly.

If only the morphology was at the origin of the chiroptical response, chirality simulations for the NPs should reproduce the measured results. We therefore used the boundary element method (BEM) to simulate the plasmonic CD (see the Experimental Section for details), taking the measured 3D shapes of the NPs as the input. The calculated *g*-factors at 532 nm are plotted as functions of the measured ones in Figure 5c. Many of the particles displayed a low *g*-factor (below 0.01) in their simulated and measured values confirming our observations for Figure 5b. However, for NPs with larger *g*-factors, no clear correlation, in terms of strength and handedness of *g*-factor, could be found, and we even observed cases where the measured values substantially deviated from the simulated ones.

All of the results presented above indicate that the chiroptical response is not only determined by obvious morphological features such as helical wrinkles. Fortunately, such well-defined features can dictate the chiroptical response (NRs batch 1), motivating continuing efforts in the synthesis of structures. However, other morphological effects also play a role, as observed for nominally achiral NPs, which can have *g*-factors of the same order as the average CD signal of chiral NRs. These nonobvious morphological chiral features are likely to strongly contribute to the high *g*-factors for the NRs in batch 2 but are hard to quantify for such complex structures and can obscure a clear chiral structure–property correlation. In addition to morphological effects, other additional parameters could be relevant. First, substrate effects were shown to have an influence on the chiroptical response.^10,21^ Even though we used immersion oil for the optical measurements of the nominally achiral gold NPs to match the substrate’s refractive index, our sample preparation is rather complex because we need to sandwich the fragile SiO_2_ TEM grid between two glass slides. Therefore, we cannot fully exclude the presence of a small air bubble behind the grid window, which could break the symmetry of the NP’s refractive environment.^10^ Furthermore, ligands on the NP’s surface could play a role as well, as they have been reported to alter the particle’s plasmonic response.^22^ In addition, we observed that the local helicity of the NR surface often changes sign within the same particle. Local helicity could couple to strong local plasmonic fields at sharp corners and edges, leading to a possible dominance of local chiral features in the chiroptical response.^23^ Although the simulation results in Figure 5c question whether this is really the case, they cannot exclude that a local plasmonic effect occurs for highly asymmetric particles with sharp local features as the presented NRs. For these cases more measurements are needed, ideally with complementary techniques, as simulations of these complex NRs are not straightforward. The reason is the high computational costs for including all local structural features as it requires input structures with a high number of surface elements. In particular, single-particle measurements resolving the whole CD spectrum and ideally decoupling absorption and scattering effects would be extremely useful to shed more light on the observed structure–property discrepancies. Additionally, studies of the CD signal under varying illumination directions could be insightful. Such measurements are cumbersome, however, and require dedicated equipment.

## Conclusion

We performed electron tomography and optical PT CD measurements of the same individual chiral NRs and nominally achiral NPs to explore the correlation of morphological and optical chirality. As opposed to what can be extracted from ensemble average measurements, we observed that each individual chiral nanorod showed different strengths and signs of the chiroptical response, with some single chiral NRs even exhibiting a more than an order of magnitude larger CD signal than that of the ensemble. For chiral NRs with strong structural helical features, the chiroptical response was in agreement with the measured morphology. However, the correlation was less obvious for less defined morphologies, even though we measured *g*-factors of similar order of magnitude. Even some single nominally achiral NPs displayed *g*-factors of the same order of magnitude. These measurements revealed that anisotropies and asymmetries, for example, in surface facets distributions, can cause “accidental” geometrical chiral features and might be responsible for the unexpected chiroptical responses. In combination with possible electromagnetic enhancements of local chiral features, these geometrical features can result in strong, although not controllable, *g*-factors.^23^ We can conclude that there is still a lot to be learned about the origin of chiroptical activity in plasmonic systems and showed that single NP correlative measurements are an important tool to do so. We propose that future measurements taking the discussed factors into account will be necessary to fully unravel the complex interplay between the morphology, plasmonic properties and chiroptical response of metallic chiral NPs.

## Experimental Section

### Wet-Chemical Synthesis of Chiral Nanorods

The faceted spherical-like AuNPs with average diameter were prepared by a seeded growth method assisted by oxidative etching.^24^ The chiral NR samples were prepared by a cosurfactant-assisted seeded growth reported previously,^17^ using single-crystalline AuNRs as seeds (batch 1: average length/diameter of 76 ± 4 nm/28 ± 3 nm, [Au(0)] = 2.4 mM; batch 2: average length/diameter of 60 ± 6 nm/11 ± 2 nm, [Au(0)] = 1.0 mM).^25^ Prior to the growth of chiral NRs, a cosurfactant solution was prepared by dissolving 1.12g of hexadecyltrimethylammonium chloride (CTAC, 99%, ACROS) and 24.9 mg of (*R*)-(+)-1,1-binaphthyl-2,2-diamine (R-BINAMINE, 99%, Aldrich) (or (*S*)-(−)-1,1-binaphthyl-2,2-diamine (S-BINAMINE, 99%, Aldrich)) in 35 mL of warm water at 50 °C. The BINAMINE-CTAC solution was then cooled down to room temperature and kept in darkness. The single-crystalline AuNRs were centrifuged (batch 1:5000 rpm, 15 min; batch 2:9000 rpm, 15 min) and washed 3 times with CTAC solution (10 mM) and another 2 times with the BINAMINE-CTAC solution, followed by overnight incubation in the BINAMINE-CTAC solution at room temperature. The growth solution of chiral NRs was prepared by mixing 200 μL of the BINAMINE-CTAC solution, 600 μL of water, and 10 μL of HAuCl_4_ solution (50 mM, Aldrich) in a 2 mL Eppendorf tube. After shaking and a rest for 5 min, the growth solution changed from pale yellow to brown, indicating the complexation of Au(III) with CTAC. Subsequently, the incubated single-crystalline AuNRs (batch 1:16 μL; batch 2:5 μL) were quickly mixed with the growth solution, followed by a fast addition of 200 μL of l-ascorbic acid (0.8 M, Aldrich). The mixture was shaken vigorously and then kept undisturbed for 15 min, during which the chiral NRs were formed. The solution was then centrifuged (batch 1:2500 rpm; batch 2:4500 rpm) for 10 min and redispersed in CTAC (1 mM). Milli-Q water (resistivity 18.2 MΩ·cm at 25 °C) was used in all experiments.

### Noncorrelative PT CD Measurements on Glass Substrates

Samples for the noncorrelative measurements were prepared by spin-coating the particles for 20 s at 500 rpm, followed by 60 s of 4000 rpm on an ozone-cleaned glass cover slide. After spin-coating, the samples were sandwiched with a cavity glass slide (Thorlabs MS15C1) to provide space for the immersion oil (*n* = 1.34 immersion oil for NRs of batch 1 and *n* = 1.51 for the highly isotropic spherical NPs). Nanoparticle-aggregates were excluded by postprocessing the measurement data with a filter function for the PT signals. The 60 nm spherical particles were measured at 5.2 mW heating power at 532 nm and 0.9 mW probe power at 780 nm. The NRs (batch 1) were measured at 2 mW heating power (660 nm) and 0.27 mW probe power (780 nm).

### Correlative Measurements

For the correlative transmission electron microscopy and optical measurement, single gold faceted NPs (diameter of 100 nm, purchased from nanoComposix) or single chiral gold NRs of batch 2 (wet chemically synthesized according to ref (17)) were drop-casted on a TEM grid with a silicon dioxide support film of 18 nm thickness and window sizes of 70 × 70 μm^2^ (Product No.: 21532–10, purchased from Ted Pella). The TEM grid was then sandwiched with a second glass slide (thickness of about 170 μm). Immersion oil was used as a photothermal medium because of its high viscosity and hydrophobicity, which were essential for the sandwiched TEM grid sample to stay stable in vertical position required in our setup. For the measurements of single gold faceted NPs, immersion oil of a refractive index of 1.51, and for the measurements of single chiral gold NRs, an immersion liquid of refractive index of 1.34 was used. TEM and optical measurements were performed on the same NPs, which were found back from pattern recognition. For the NRs and faceted sphere-like NP measurements, the heating laser powers were 25.6 mW (660 nm) and 8 mW (532 nm), respectively, and the illuminated area was about 20 μm^2^. The probe laser had powers of 0.8 mW and 2 mW (780 nm), respectively, and was focused to a diffraction-limited area. Please note that the illumination area of the heating beam was ∼3× lower in the uncorrelated measurements compared to the ones in the correlated measurements.

### *g*-Factor Analysis of Optical Measurements

The CD *g*-factor, which is defined as
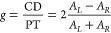
was calculated for each particle as the ratio of mean values of photothermal and circular dichroism signals averaging the signal in an area of 7 × 7 pixels^2^ around the peak position of each individual particle within the scanned images. We first used a peak finding function to find the peaks in the PT images and then used a cross correlation to calculate the center position of the particles in the CD images (using absolute values).

### Transmission Electron Microscopy and Tomography

HAADF-STEM images and electron tomography tilt series were acquired using a FEI-Osiris electron microscope operated at 200 kV. Tilt series were acquired for the maximal possible tilt range for the SiO_2_ TEM grids, which was generally around ±65°. For the non-correlative measurements for NRs of batch 1, we used a standard carbon TEM grid, allowing for a tilt range of ±75°. The tilt increment was 3° in both cases. The tilt series for the achiral nanoparticles were reconstructed in the following manner: after aligning the tilt series images by cross-correlation and correcting for cupping artifacts, the stacks of aligned projection images served as inputs for 100 iterations of the expectation maximization implemented in the ASTRA toolbox.^26,27^ The reconstructions were furthermore thresholded using the Otsu method and the thresholded reconstructions were used for determining the volume. The tilt series for the more complex chiral NRs were reconstructed in the following manner: after distortion corrections with the help of a convolutional neural network, 3D reconstructions were performed by the simultaneous iterative reconstruction technique (SIRT) and the application of thresholds in real and Fourier space in an iterative process (20 total iterations with 25 SIRT iterations per step), following our previous work.^17^

### Quantification of Asphericity for Faceted NPs

The asphericity is a metric used to determine how much a given shape differs from a sphere. To calculate this metric, we segmented the 3D ET reconstructions to retrieve a binary volume. We then calculated the volume of the reconstructed shape and created a new binary volume containing a perfect sphere with the same volume. The asphericity was then calculated as the minimal shape error between the reconstructed volume and the sphere.

### Geometrical Chirality Quantification of the Quasi-Spherical NPs

The Hausdorff chirality measure is a measure to quantify the chirality of a set of points which typically describe a molecule. This chirality measure is determined by calculating the minimum Hausdorff distance between a set of points and the mirror image of that set of points. To make the measure scale-invariant, the result is then divided by the diameter of this set of points, which is calculated as the maximum distance between any two points of the set. However, since our data is stored as a 3D volume, we adapted the Hausdorff method as follows and refer to it as geometrical chirality. First the 3D ET reconstructions were segmented into binary volumes. Next, analogously to the Hausdorff chirality measure, we define a chirality measure as the minimum shape error between the binary reconstruction and its mirror image. The shape error is defined as the number of mismatching voxels divided by the number of voxels included in the volume and is therefore already scale-invariant. The chirality measure is found by minimizing the shape error for rigid transformations of the mirrored volume, thereby taking all possible mirror axes into account.

### Boundary Element Method Simulations

The discretized Otsu thresholded tomography reconstructions of the Au nanoparticles (downsampled to 2000 surface elements for computational feasibility) served as an input for BEM calculations, which were performed solving the full Maxwell equations at 100 points in the wavelength range from 500 to 700 nm using left and right circularly polarized light using the MNPBEM Matlab toolbox v17.^28^ The propagation direction of light was along [100], which best mimics the PT CD experiments as it corresponds to the direction perpendicular to the orientation of the NPs on the TEM grid (determined from ET). The *g*-factor was then determined as defined in our optical measurements and earlier work.^15^

### Quantification of Helicity

The helicity quantification was performed following the procedure explained in ref (18) using the HeliQ Python package. More specifically, the 3D gradient of the electron tomography reconstructions was calculated for each particle after alignment with the helical axis. The gradient magnitude is largest where the reconstruction intensity changes, that is, at the surface of a NP, and was therefore used to identify the surface. The orientation of the gradient was then used to find the inclination angle of the surface in each voxel. This combined information is used to quantify the helicity of a nanoparticle in two distinct ways.

#### Helicity Function

**Figure 6:**
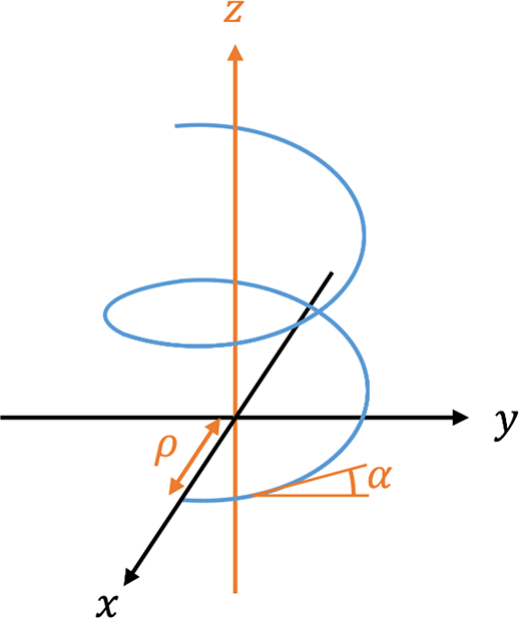
Illustration of the parameters ρ and α. In blue is an example of the helical structure winding around the *z*-axis (long axis). ρ is the distance from a certain helical feature to the long axis and α is its inclination angle with respect to the *x*–*y* plane (plane orthogonal to the long axis).

The helicity function is a 2D distribution that indicates the difference between the presence of left-handed (negative) and right-handed (positive) inclined surface features. It is calculated using
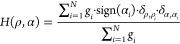
where *g*_*i*_ is the gradient magnitude in voxel *i* of the electron tomography reconstruction, α_*i*_ is the inclination angle in that voxel, and ρ_*i*_ is the distance between voxel *i* and the helical axis. ρ and α are, respectively, plotted on the *y*- and *x*-axis of the helicity function plot. The physical meaning of these parameters is illustrated in Figure 6.

#### Helicity Map

The helicity map shows the distribution of left-handed (negative, blue) and right-handed (positive, red) features across the surface of a nanoparticle. It is calculated as
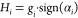
after which a Gaussian filter was applied to reduce noise and artifacts in the result.

## Supporting Information Available

Ensemble CD spectra of chiral nanorods; Structural and optical analysis of batch 1; 2D HAADF-STEM images of batch 2; Helicity functions of chiral nanorods from batch 2 and influence of single wavelength measurements; Comparison of CD of 100 nm faceted sphere-like and 60 nm spherical gold nanoparticles; Electron tomography of faceted sphere-like gold NPs; Histograms of volumes of gold nanoparticles obtained from electron tomography; Effect of defocusing on the CD *g*-factor; Sign of the CD signal in the optical measurements (PDF)
