# Supplementary material for: Photothermal Circular Dichroism Measurements of Single Chiral Gold Nanoparticles Correlated with Electron Tomography
Source: ACS Photonics. 2022 Nov 8;9(12):3995–4004. doi: 10.1021/acsphotonics.2c01457 (PMC9783064; doi:10.1021/acsphotonics.2c01457)
Supplement: Supplementary file 1 — ph2c01457_si_001.pdf [file ph2c01457_si_001.pdf]

Supporting Information: Photothermal Circular Dichroism Measurements of Single Chiral Gold Nanoparticles Correlated with Electron Tomography

Patrick Spaeth,[†,‡,¶] Subhasis Adhikari,[†,‡] Wouter Heyvaert,[§] Xiaolu Zhuo,[‖,⊥,#] Isabel García,[‖,⊥] Luis M. Liz-Marzán,[‖,⊥,@] Sara Bals,[§] Michel Orrit,[*,†] and Wiebke Albrecht[*,¶,§,†]

†*Huygens-Kamerlingh Onnes Laboratory, Leiden University, 2300 RA Leiden, The Netherlands*

‡*These authors contributed equally.*

¶*Department of Sustainable Energy Materials, AMOLF, Science Park 104, 1098 XG Amsterdam, The Netherlands*

§*EMAT and NANOlab Center of Excellence, University of Antwerp, Groenenborgerlaan 171, B-2020 Antwerp, Belgium*

‖*CIC biomaGUNE, Basque Research and Technology Alliance (BRTA), Paseo de Miramón 182, 20014 Donostia-San Sebastián, Spain*

⊥*CIBER de Bioingeniería, Biomateriales y Nanomedicina (CIBER-BBN), Paseo de Miramón 182, 20014 Donostia-San Sebastián, Spain*

#*School of Science Engineering, The Chinese University of Hong Kong (Shenzhen), Shenzhen 518172, China*

@*Ikerbasque (Basque Foundation for Science), 48009 Bilbao, Spain*

E-mail: orrit@physics.leidenuniv.nl; w.albrecht@amolf.nl

Ensemble CD spectra of chiral nanorods

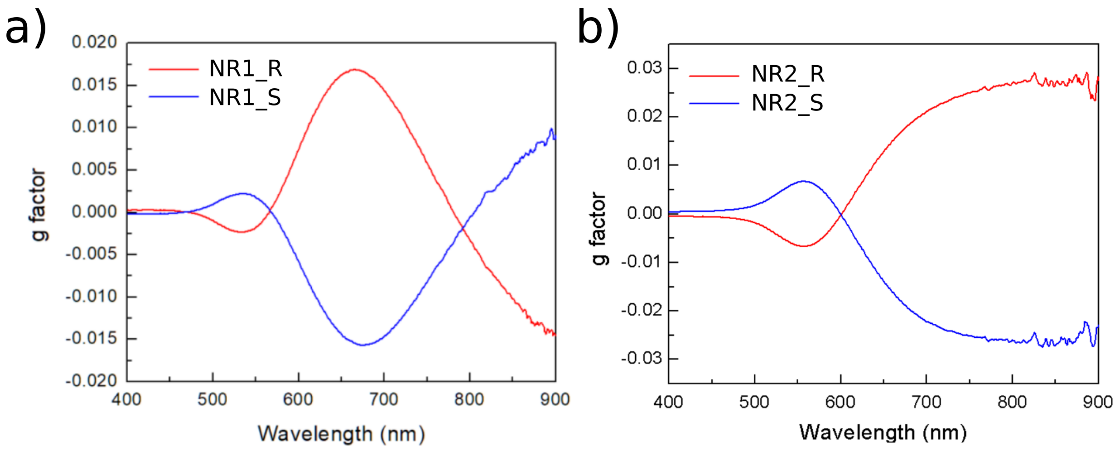

Figure S1: Ensemble circular dichroism (CD) spectra of an aqueous suspension of chiral gold nanorods of a) batch 1 and b) batch 2.

Figure S1 shows a CD spectrum of a diluted stock suspension of chiral nanorods (batch 1 and batch 2), obtained with a JASCO J-1500 CD spectrometer. Our optical single-particle CD measurements were carried out at 660 nm at which the ensemble CD g-factor is about ± 0.015 for the R and S version, respectively, of the particles from batch 1 and batch 2.

Structural and optical analysis of batch 1

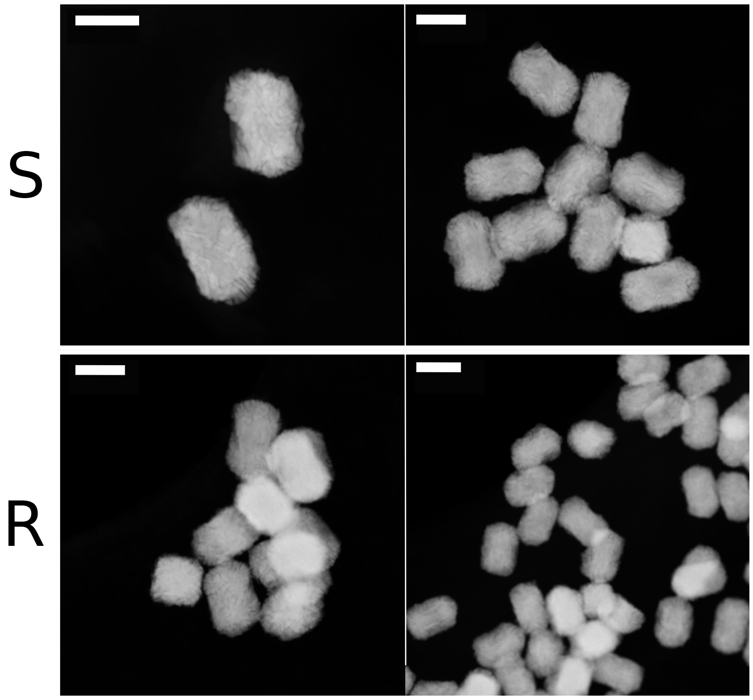

Figure S2: 2D HAADF-STEM images of chiral NRs of batch 1. Top panels show the S version of the rods and lower panels show the R version of the rods. Scale bars are 70 nm in each image.

Representative 2D HAADF-STEM images of NRs of both handedness samples of batch 1 are shown in Figure S2. Figure S3 shows orthoslices through representative electron tomography reconstructions from particles of batch 1 (from the S enantiomer shown in Figure 1b) and batch 2 (P1 and P2 from Figure 3a and c). It is evident that the particles from batch 1 have much finer wrinkles and more repetitive patterns as indicated by the arrows, which have previously been identified as the origin of strong chiroptical signals in these systems (references 17 and 18 in the main text).

Figure S4 shows optical measurements of the absorption (PT) and the circular dichroism (CD) of chiral NRs of batch 1 spincoated on a glass cover slip. The CD scans of the S and R samples (top and bottom) show a strong bias for each handedness, i.e. for one sign. Particles not encircled are probably clusters and were excluded from the analysis.

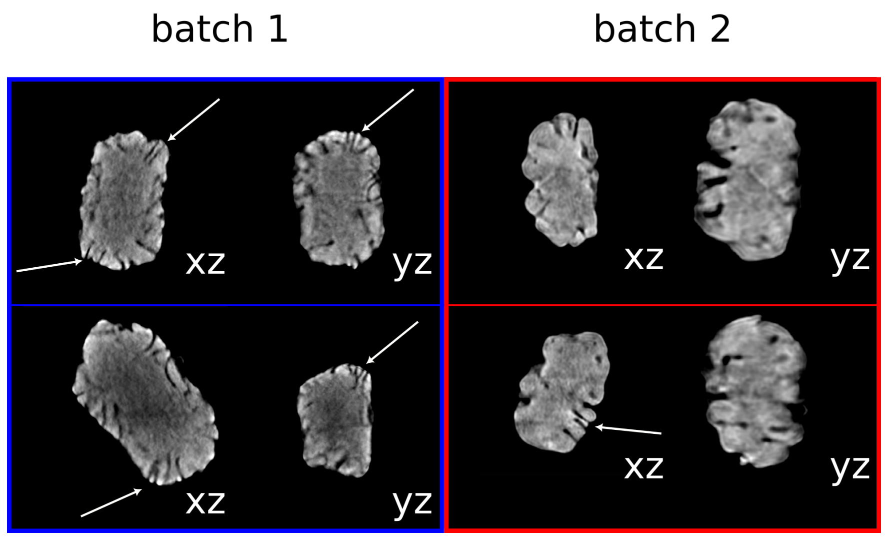

Figure S3: Orthoslices through some chiral nanorods from batch 1 (left) and batch 2 (right). Arrows indicate regions that contain repetitive wrinkels (helical features).

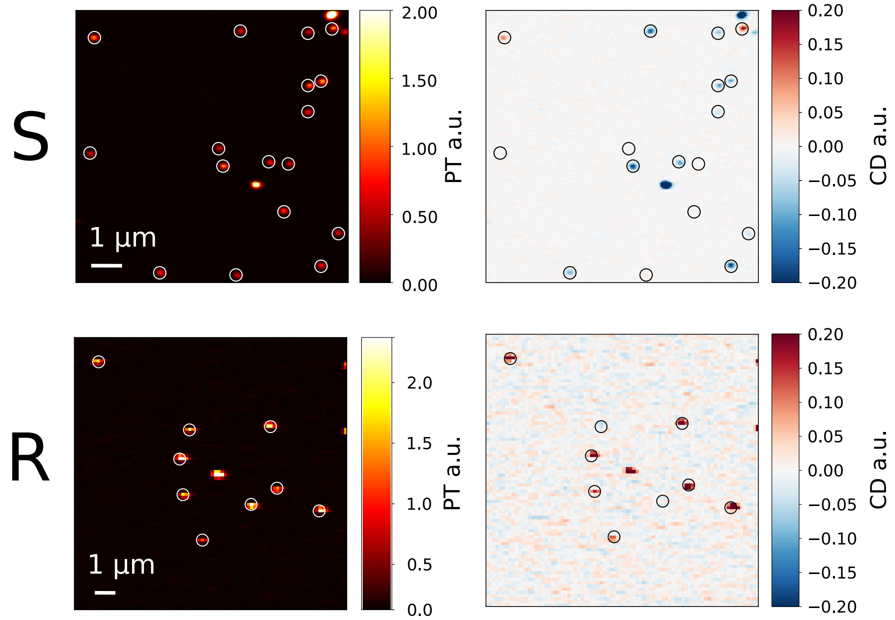

Figure S4: Photothermal (PT) and circular dichroism scans of chiral gold NRs of batch 1. The left and right plots show the PT and CD signals, respectively. Upper panels display scans from the S enantiomer sample of the NRs and the lower panels show the R enantiomer sample. Scale bars are indicated in the inserts. Circles are guides for the eye. Particles not encircled are probably clusters of more than one particle as judged from the higher PT signal, which scales with volume.

2D HAADF-STEM images of batch 2

2D HAADF-STEM images of 23 examples of single chiral gold nanorods of batch 2 and two examples of dimers are shown in Figure S5. For all of those 25 particles (single particles and

dimers) we performed correlative measurements and the HAADF-STEM images are shown in the order of decreasing g-factors (strong positive to strong negative). The CD data for the single particles are included in the histogram of Figure 2 of the main text.

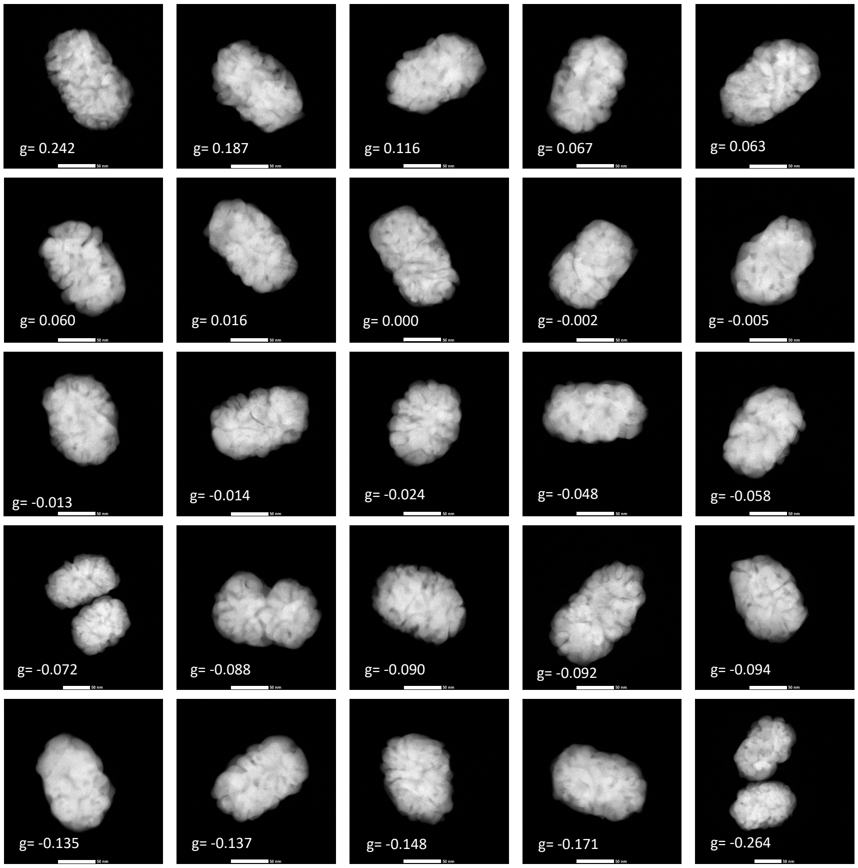

Figure S5: 2D HAADF-STEM images of a total of 25 chiral gold nanorods and dimers of nanorods from batch 2, arranged by decreasing g-factors, mentioned in the inset. Scale bars are 50 nm.

Helicity functions of chiral nanorods from batch 2 and influence of single wavelength measurements

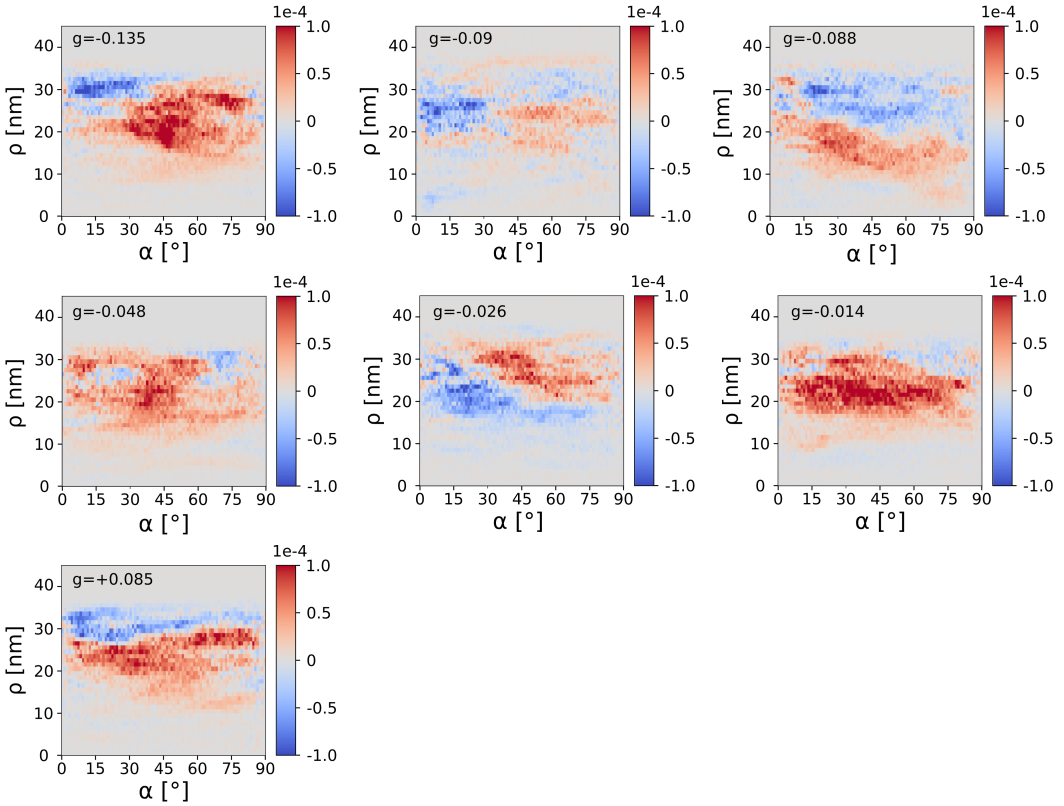

Figure S6: Helicity functions of chiral nanorods from batch 2. Their correlated optical g-factor is given in the inset. As in Figures 1 and 3 in the main text, the x and y axes correspond to the radius ρ and inclination angle α.

Figure S6 shows the results of the correlated optical-ET measurements of 7 NRs of batch 2. The helicity functions obtained from the ET reconstructions and the corresponding optical CD g-factors (at 660 nm) are shown.

One may argue that the large spread in g-factors and non-obvious correlation of the chiroptical activity and the structural helicity is dominated by plasmonic spectral shifts, which we do not account for as we only measure at one wavelength (660 nm). In other words, individual particles might have strongly different absorption and CD spectra. We believe, however, that this is not the main cause for the observed discrepancy for batch

2. First, if this was the case, more random CD sign switches could also be expected, which should also occur for the measurements of NRs of batch 1, which we did not observe. Second, we can estimate the differences in peak positions from a correlative analysis of the particles' PT and CD signals and their g-factor values.

If the absorption (PT) and CD peaks would be shifted a lot between different particles, we would expect a large spread in PT values and a decrease in g-factor strength for decreasing PT signals but no correlation between the CD strength and the corresponding g-factor. Figure S7a shows the histogram of PT signals of the measured NRs from batch 2. The width of the histogram ($\sim$ factor of 3) might appear large, but considering that the PT signal scales with the volume (V $\sim$ l*d^2, l = length, d = diameter), to first approximation (if plasmonic effects are absent or evenly strong for all particles), it can be explained by a reasonable size variation of the particles ($\pm$ 20% in both dimensions (l,d)). Moreover, when we measured the particles we performed large area scans containing many particles. Consequently, the bent surface of the TEM grid resulted in some particles being partially out of focus, which also contributes to the spread. As discussed below, this defocus did not influence the g-factor analysis.

From Figure S7b we also find that there is no correlation between the PT signal and its g-factor. More importantly in c) we show the correlation plot between the CD signal (which depends on the size and the strength of the differential absorption ($\sim$ A_l-A_r *V, V = volume) and the g-factor which only depends on the relative strength of the differential absorption ($\sim A_l$-A_r)/(A_l+A_r). If every particle has a similar absorption spectrum, then the average absorption (A_l+A_r) scales linearly with V and thus g scales linearly with CD. The clear linear correlation in Figure S7c thus shows that the differences between the spectra cannot be not too large.

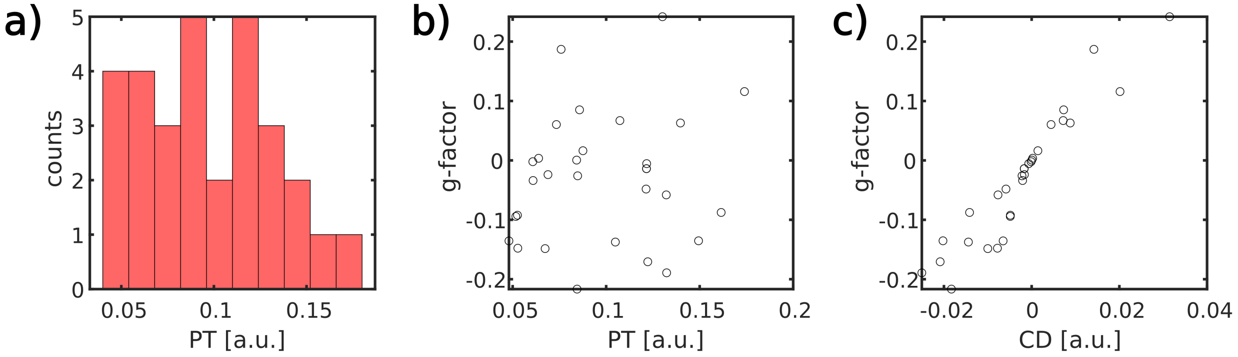

Figure S7: (a) Histogram of PT signals of chiral nanorods from batch 2 (all single particles verified by TEM). (b) correlation plot of g-factor and PT signal. (c) correlation of g-factor and CD signal.

Comparison of CD of 100 nm faceted sphere-like and 60 nm spherical gold nanoparticles

Figure S8 shows (S)TEM images of the 100 nm (top) and 60 nm (bottom) particles. The HAADF-STEM images show that the 100 nm particles mostly display distinct facets and a homogeneous contrast, which can be attributed to their single crystallinity. The 60 nm particles on the other hand are much rounder (spherical) as a consequence of the oxidative etching step in their synthesis procedure.

Figure S9 shows the histograms of g-factors of 100 nm faceted sphere-like gold nanoparticles (green) and 60 nm spherical gold nanoparticles (blue).

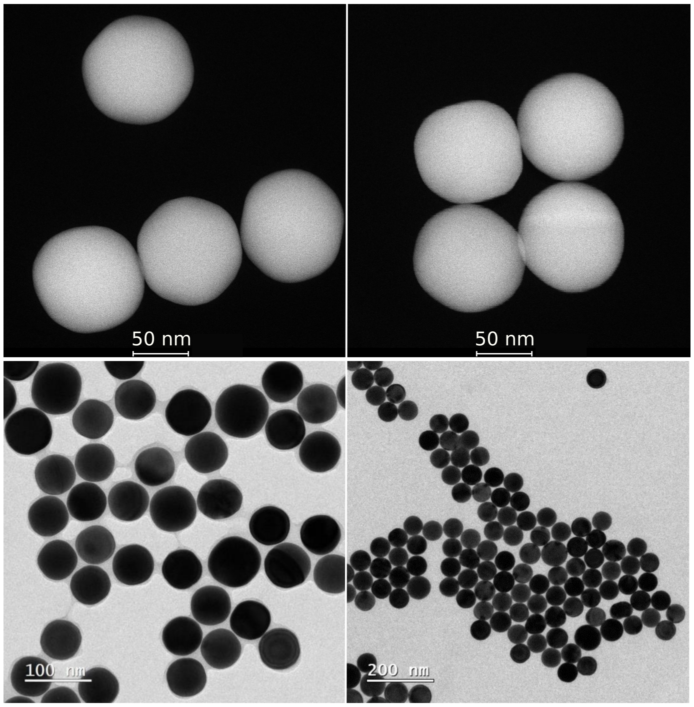

Figure S8: The top row shows HAADF-STEM images of the faceted 100 nm gold nanoparticles purchased from nanoComposix. The lower row shows bright-field TEM images of the 60 nm spherical gold nanoparticles.

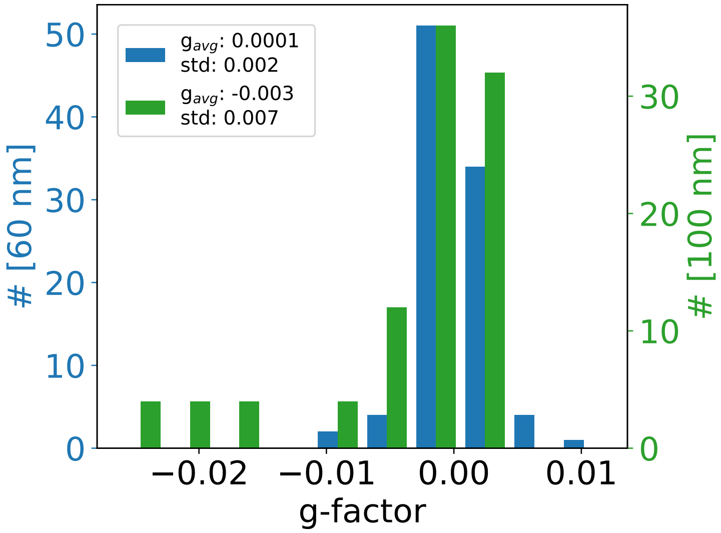

Figure S9: Histogram of CD g-factors of 100 nm faceted sphere-like gold nanoparticles purchased from nanoComposix (green) and 60 nm spherical particles synthesized by us (blue).

Electron tomography of faceted sphere-like gold NPs

Electron tomography reconstruction visualizations of the four single gold nanospheres mentioned in the main text in Figure 4, are shown in Figure S10 in three orthogonal planes, XY,

XZ and YZ.

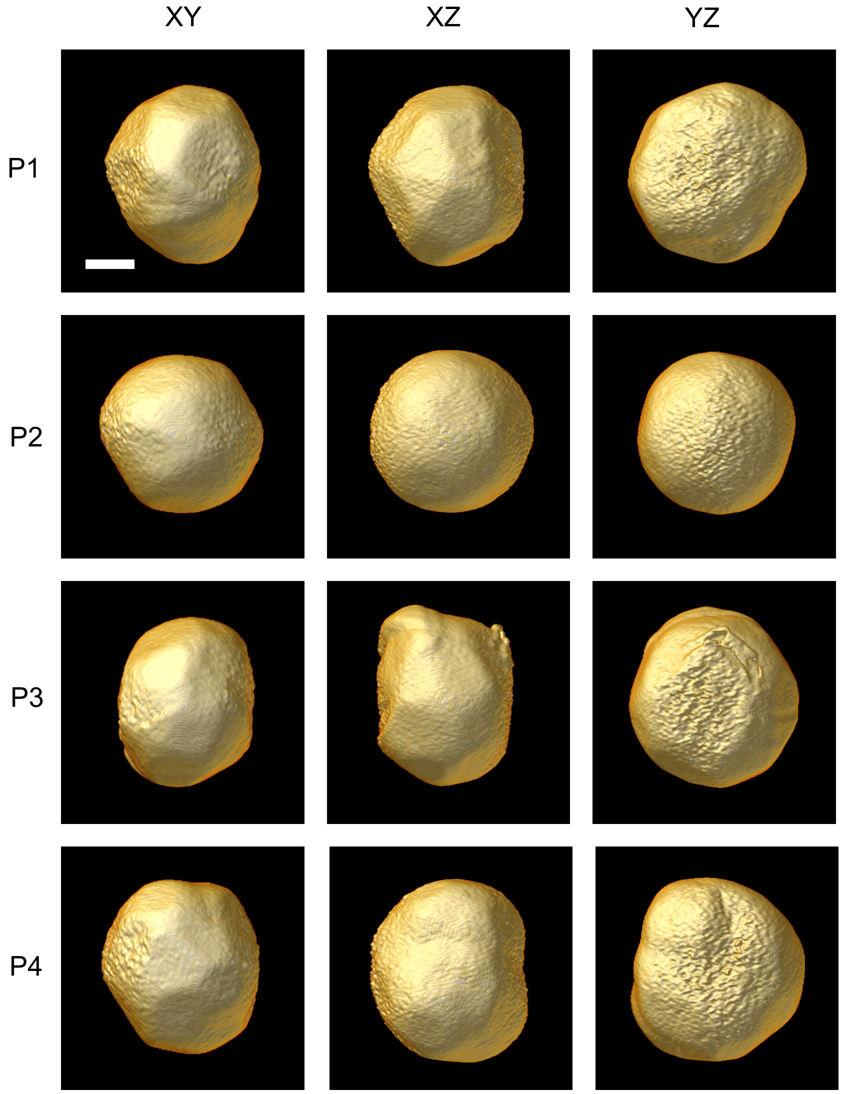

Figure S10: Electron tomography images of four single gold nanospheres in three orthogonal planes, XY, XZ and YZ. The same four particles are also shown in the main text in Figure 4 but only in the YZ plane. Scale bar is 30 nm.

Histograms of volumes of gold nanoparticles obtained from electron tomography

Volumes of single faceted 100 nm sphere-like gold nanoparticles were calculated from the electron tomography reconstructions. The histogram of volumes of 25 single nanoparticles is shown in Figure S11. The histogram indicates that the particles are indeed uniform in size as claimed by the provider nanoComposix.

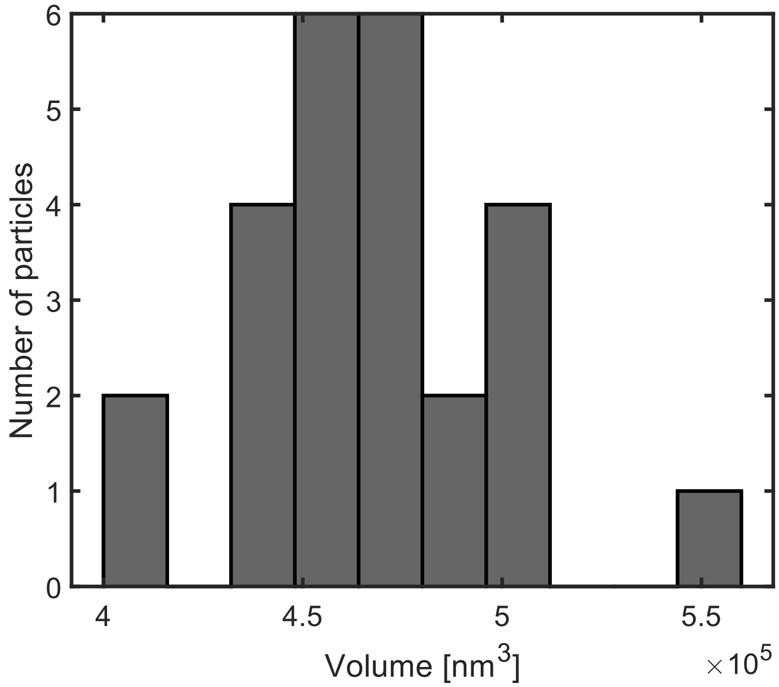

Figure S11: Histogram of volumes of a total of 25 single gold nanoparticles obtained from electron tomography, showing a rather uniform size distribution.

Effect of defocusing on the CD g-factor

Because of the slightly bent surface of the TEM grid windows, some particles are in focus and others are slightly out-of-focus in our optical measurements as shown in Figure 4 in the main text. Therefore, we checked if the slight defocusing may have an influence on

the CD g-factor. We measured a single 100 nm gold nanoparticle in-focus and out-of-focus as shown in Figure S12. Slight defocusing decreases both photothermal (PT) and circular dichroism (CD) signals alike and therefore the calculated g-factor (*i.e.* the ratio of CD and PT) remains nearly unchanged. Therefore we conclude that the slight defocusing does not have any influence on the calculated g-factors.

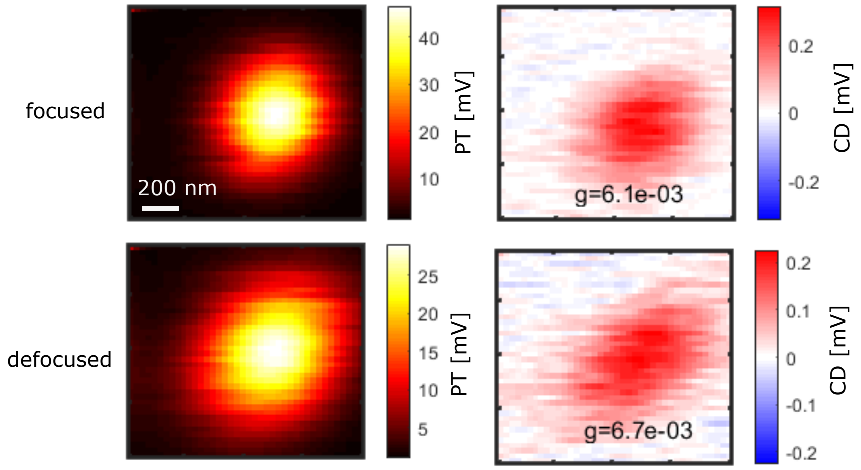

Figure S12: Photothermal (PT) and circular dichroism (CD) image of a single gold nanoparticle in (a) focused and (b) slightly defocused conditions showing slight defocusing has nearly no influence on the value of the g-factor.

Sign of the CD signal in the optical measurements

We realized that the sign of the CD signal depends on the settings of the quarter wave-plate and the lock-in amplifier of our setup. For the measurements of NRs batch 1, we recorded all required parameters and are absolutely certain of the (absolute) sign. The sign we determined from the signle particle measurements also corresponds well to the CD ensemble sign at 660 nm. For batch 2 and the faceted NPs, however, we are uncertain about these parameters. That means that we cannot unambiguously tell whether a negative/positive CD signal refers to more absorption of left- or right-circularly polarized light for these measurements. However, we ascertained that the measurements are sign-consistent within themselves, or in

other words, that all particles that show a certain sign in the CD measurement, all absorb either more left- or right-circularly polarized light. In addition, as we saw for batch 1 that the average g-factor corresponds well to the CD ensemble average, we attributed the signs for batch 2 in the same way, i.e. the average of the CD g factor histogram was in accordance with the value of the ensemble CD at 660 nm. In any case, the conclusions drawn for batch 2 are not dependent on the sign and are valid even if all signs were swapped.
